# High-Grade Glioma of the Ventrolateral Medulla in an Adult: Case Presentation and Discussion of Surgical Considerations

**DOI:** 10.1155/2016/6813089

**Published:** 2016-05-08

**Authors:** Angela Spurgeon, Viet Le, Sanjay Konakondla, Douglas C. Miller, Tamera Hopkins, N. Scott Litofsky

**Affiliations:** ^1^Division of Neurosurgery, University of Missouri School of Medicine, Columbia, MO 65212, USA; ^2^University of Missouri School of Medicine, Columbia, MO 65212, USA; ^3^Department of Pathology and Anatomical Sciences, University of Missouri School of Medicine, Columbia, MO 65212, USA; ^4^Division of Hematology Oncology, University of Missouri School of Medicine, Columbia, MO 65212, USA

## Abstract

*Background*. High-grade gliomas of the brainstem are rare in adults and are particularly rare in the anterolateral medulla. We describe an illustrative case and discuss the diagnostic and treatment issues associated with a tumor in this location, including differential diagnosis, anatomical considerations for options for surgical management, multimodality treatment, and prognosis.* Case Description*. A 69-year-old woman presented with a 3-week history of progressive right lower extremity weakness. She underwent an open biopsy via a far lateral approach with partial condylectomy, which revealed a glioblastoma. Concurrent temozolomide and radiation were completed; however, she elected to stop her chemotherapy after 5.5 weeks of treatment. She succumbed to her disease 11 months after diagnosis.* Conclusions*. Biopsy can be performed relatively safely to provide definitive diagnosis to guide treatment, but long-term prognosis is poor.

## 1. Introduction

Glioblastomas (GBM) account for 54% of CNS gliomas; however the incidence of glioblastoma in the brainstem is not well defined [1]. In a series of 21 patients with gliomas, Hundsberger et al. [2] found six (28.6%) brainstem glioblastomas of which two originated in the medulla. In a larger series Kesari et al. [3] had found only 3 (5.5%) glioblastomas out of 54 surgically sampled brainstem gliomas.

Due to the rarity of medullary brainstem glioblastomas, their diagnosis and management are complex and controversial. We report the case of a 69-year-old woman with a glioblastoma of the ventrolateral medulla to highlight differential diagnosis, anatomical considerations relating to options for surgical management, and multimodality treatment.

## 2. Case Presentation

### 2.1. History and Examination

**Figure 1:**
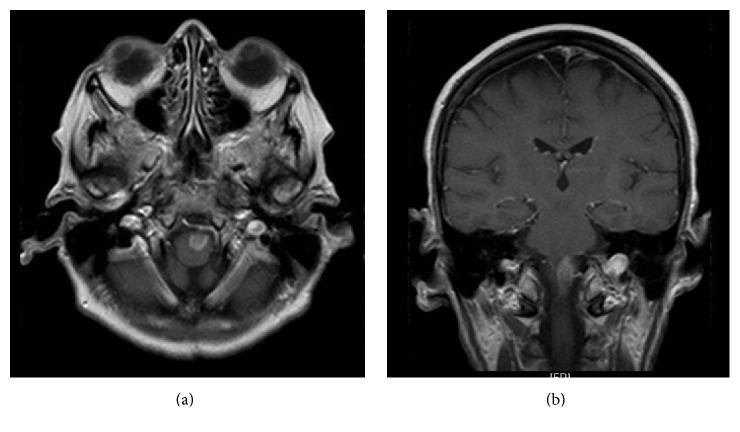
Initial brain MRI. (a) Axial T1-weighted image with gadolinium, showing the enhancing left medullary lesion. (b) Coronal T1-weighted image with gadolinium showing the same medullary lesion.

A 69-year-old Caucasian woman presented to an outside hospital with a 3-week history of progressive right lower extremity weakness. An initial MRI demonstrated a 1.9 × 0.8 × 1.0 cm contrast-enhancing mass in the left ventrolateral aspect of the medulla (Figure 1). She was diagnosed with a “stroke” at the outside hospital and transferred to an inpatient rehabilitation facility. Two weeks later she developed drooling with slurred speech and was transferred to the University of Missouri Hospital and Clinic's Neurosurgery Service. Physical examination revealed a left hypoglossal nerve palsy but a right accessory nerve weakness. Motor examination demonstrated full strength on the left side, with right-sided weakness (deltoids, biceps, and triceps 4/5, grip 2/5, hip flexor and knee extensors 4/5, knee flexor, and dorsiflexion and plantar flexion 0/5).

### 2.2. Diagnosis

**Figure 2:**
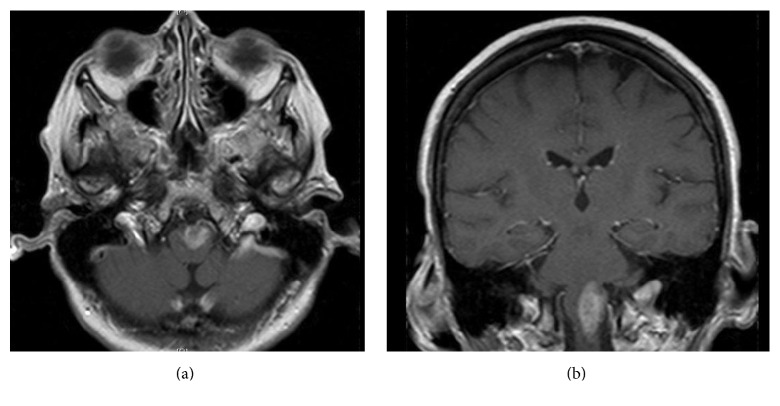
Brain MRI 2 weeks after the initial MRI. (a) Axial T1-weighted image with gadolinium, showing the enhancing left medullary lesion increased in size. (b) Coronal T1-weighted image with gadolinium showing the same medullary lesion increased in size.

A repeat MRI revealed that the mass was larger, now 3.2 × 1.1 × 1.4 cm (Figure 2). The differential diagnosis (Table 1) was quite extensive, but lymphoma and glioma were favored on the basis of history and imaging. Further metastatic workup and a lumbar puncture were negative.

**Figure 3:**
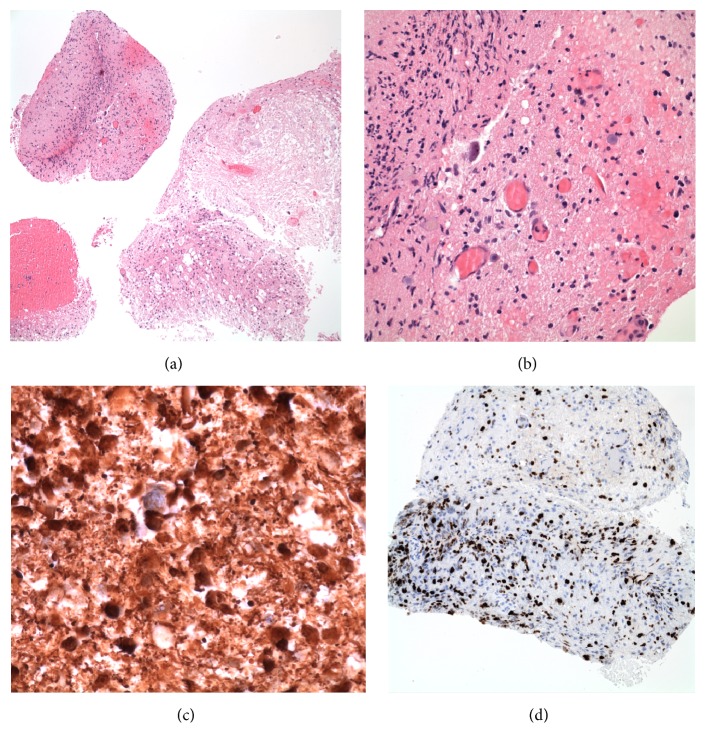
Histopathology of medullary tumor. (a) Multiple small fragments of the biopsy include one with significant necrosis, one with relatively high cell density, and one with considerable hemorrhage. H&E, original magnification 100x. (b) At high magnification, this portion of the biopsy shows tumor with moderate cell density bordering necrotic tumor. H&E, original magnification 400x. (c) Many of the tumor cells have cytoplasmic GFAP immunoreactivity, establishing their astrocytomatous phenotype. Original magnification 600x. (d) A Ki67 immunostain shows a proliferative index of up to about 25%, consistent with the diagnosis of a high-grade glioma. Original magnification 200x.

Surgical intervention was planned to establish a definitive diagnosis and achieve brainstem decompression if possible. The medullary lesion was biopsied using neuronavigation with BrainLAB via a far lateral approach with partial condylectomy. Intraoperative monitoring was utilized to observe the integrity of neural pathways and included somatosensory evoked potentials (SSEPs), brainstem auditory evoked potentials (BAEPs), motor evoked potentials (MEPs), and electromyography (EMG) for the lower cranial nerves. The lesion was quite firm and rubbery but blended in with surrounding tissues. Based on an intraoperative frozen section report of high-grade glioma, the texture of the lesion, and a temporary loss of MEPs during the procedure, resection was limited. A diagnosis of glioblastoma (WHO grade IV) was subsequently confirmed by the final pathology examination (Figures 3(a)–3(d)).

### 2.3. Hospital Course

The patient's postsurgical course was complicated by aspiration pneumonia requiring a period of reintubation. Postoperative hoarseness led to a diagnosis of left vocal cord paralysis. Her right-sided weakness remained unchanged from her preoperative baseline. On postoperative day five, she was transferred to a hospital closer to home for continued acute care and adjuvant therapy.

### 2.4. Treatment

**Figure 4:**
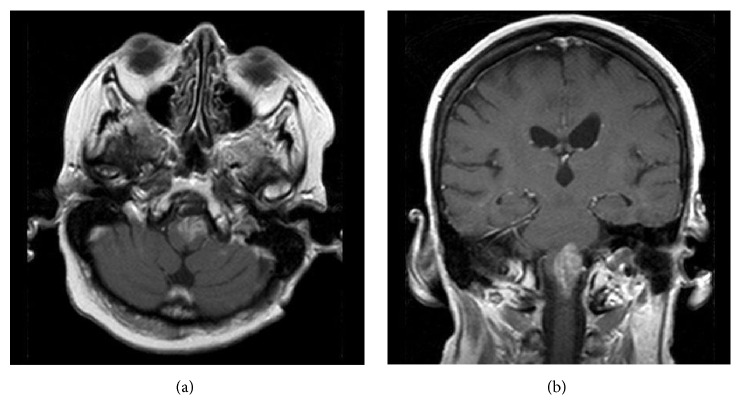
Brain MRI 10 months after surgery. (a) Axial T1-weighted image with gadolinium, showing progression of the enhancing left medullary lesion. (b) Coronal T1-weighted image with gadolinium showing the same medullary lesion.

Treatment was initiated with temozolomide 100 mg/day. A concurrent course of radiation therapy (total 5600 cGy divided over 28 fractions) was completed. She achieved some improvement in lower extremity function so that she was able to stand and pivot. She was hospitalized multiple times during radiotherapy for recurrent UTIs and required pharmacologic treatment for depression. She elected to stop her chemotherapy after 5.5 weeks of daily temozolomide. Recurrent headaches 10 months after surgery prompted a repeat MRI which demonstrated disease progression (Figure 4). The patient and her family elected to proceed with palliative care. She succumbed to her disease 11 months after diagnosis.

## 3. Discussion

Glioblastoma of the medulla is rare; thus the ability to draw definitive conclusions from the literature can be a challenge. Most larger series combine all gliomas of the midbrain, pons, and medulla together making it difficult to draw specific conclusions with regard to GBM of the medulla. Additionally tumors of the medulla vary with regard to location (ventrolateral, ventral, diffuse, and exophytic dorsal) which affects surgical decision-making and multimodality therapies.  Table 2 summarizes case reports of documented medullary adult high-grade gliomas in the literature. The remainder of the discussion will focus on the larger body of brainstem glioma literature with emphasis placed on medullary lesions as much as possible.

### 3.1. Clinical Features and Differential Diagnosis

The signs and symptoms of a brainstem high-grade glioma overlap with those of many other CNS diseases and are dependent on the location of the lesion. Clinical features observed at diagnosis may include gait disorders, visual disturbances, limb weakness, and cranial nerve deficits [3, 13, 14]. The differential diagnosis of a brainstem mass is extensive (Table 1) [7, 15–20].

MRI is currently an essential, noninvasive tool for evaluating brainstem lesions. While imaging characteristics can help narrow the differential diagnosis, multiple studies [11, 21–24] demonstrate disparity between the MRI-based diagnoses and histopathological diagnoses [11, 21–24]. Tissue confirmation to correctly diagnose and adequately treat brainstem lesions is often necessary. Our patient was eager to have a definitive diagnosis to plan for treatment and prognosis.

### 3.2. Anatomical Considerations in relation to Surgical Treatment

The medulla oblongata extends from the inferior pontine sulcus to the roots of C1. Anteriorly, the medulla has three longitudinal fissures. The pyramids are elevated structures on either side of the anterior median fissure and comprise the descending corticospinal tracts. The paramedian sulci, also known as the anterolateral sulci or preolivary sulci, are situated medial to the olives. The rootlets of the hypoglossal nerves exit from the preolivary sulci while the rootlets of the accessory, vagus, and glossopharyngeal nerves exit from the postolivary sulci [25].

Internal structures at this level consist of the dorsal vagus, hypoglossal, ambiguus, and inferior olivary nuclei and the nuclei of the solitary tracts. The autonomic centers participating in swallowing, respiratory, and cardiovascular functions are located in and around the reticular formation in the upper half of the dorsal medulla. The medial lemniscus originates near the caudal edge of the fourth ventricle floor and runs upward on either side of the midline, eventually coursing posterior to the pyramidal tract as it enters the pons. The pyramidal tract is found in the most anterior part of the medulla while the medial longitudinal fasciculus (MLF) runs under the posterior (ventricular) surface just lateral to the median sulcus.

Suggested safe neurosurgical entry points for open procedures into the ventrolateral medulla include the preolivary (anterolateral) sulcus located between the caudal roots of the hypoglossal and the rostral C1 rootlets [26]. Some neurosurgeons recommend this only for exophytic lesions due to its close proximity to the pyramidal tract and its decussation [27]. The postolivary sulcus, between the olive and inferior cerebellar peduncle, is ventral to the vagal and glossopharyngeal rootlets and represents another entry point. The nucleus ambiguus is generally encountered 0.4 cm below the postolivary sulcus [26, 28].

**Figure 5:**
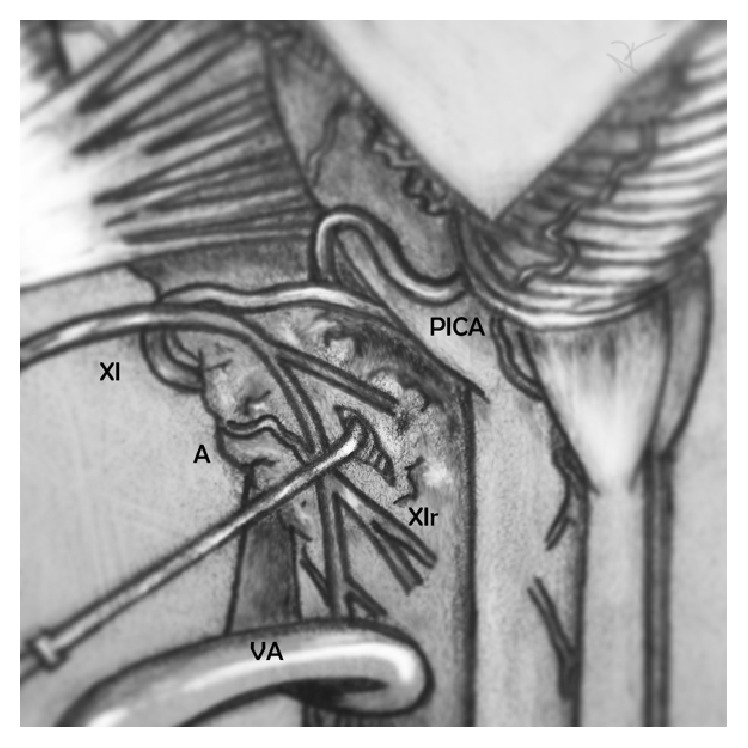
Intraoperative illustration detailing the surgical corridor from a far lateral approach. XI: cranial nerve 11; A: abnormality; VA: vertebral artery; XIr: cranial nerve 11 rootlet; PICA: posterior inferior cerebellar artery.

We opted to create a corridor between several rootlets of the accessory nerve and a bulbous region in the ventrolateral medulla near the postolivary sulcus (Figure 5). The patient's difficulty postoperatively with swallowing and her left vocal cord paralysis were likely related to infiltration of the lesion into the nucleus ambiguus. Preoperative vocal cord paralysis was not fully assessed but was likely present to some degree.

### 3.3. Surgical Considerations: Type of Surgery

Biopsy is recommended and allows the advantage of histopathologic diagnosis and molecular genetic analysis. Generally, stereotactic biopsies can be performed safely with high diagnostic success rates. Kickingereder et al. [29] completed a large meta-analysis of 1480 stereotactic biopsy procedures for brainstem tumors (including only 4 pure medullary tumors and 9 tumors involving both the pons and medulla). They found a diagnostic success of 96%, 7.8% overall morbidity, 1.7% permanent morbidity, and 0.9% mortality. Furthermore, subgroup analysis of biopsy trajectory, imaging modality used for biopsy planning, and tumor location failed to reveal a significant influence on outcome measures.

A number of other approaches, including ipsilateral transfrontal, contralateral transfrontal, and transcerebellar, are available for the stereotactic biopsy of medullary brainstem lesions. Each approach has advantages and disadvantages and the trajectory must be tailored to the lesion. The contralateral or ipsilateral transfrontal approaches have been shown to be effective in the biopsy of medullary lesions [12, 23, 30–32]. The tentorium may limit the accessibility of midline medullary abnormalities and the biopsy trajectory will have to be planned accordingly. The ventricular system should be avoided, not only to minimize the risk of hemorrhage, but also to prevent CSF loss and target shift. A contralateral transfrontal approach may be necessary to avoid the ventricular system when accessing laterally positioned lesions [30]. Procedures performed with only local anesthesia have been described [9, 33]. The suboccipital transcerebellar approach [32, 34] provides the shortest trajectory for accessing lesions of the lower midbrain, pons, and rostral medulla. Positioning often requires general anesthesia; however awake procedures have been described [34, 35].

Recent advances in neuroimaging, navigation technology, microsurgery, and anatomic knowledge have changed how we classify brainstem gliomas [36–39] and have helped identify surgically resectable lesions. Many studies advocate surgical resection of well-demarcated posteriorly, posterolaterally, and ventrolaterally located tumors in patients with relatively mild neurological symptoms [10, 36, 40–43].

Dey et al. [44] examined 240 patients from the Surveillance, Epidemiology and End Results (SEER) database with WHO grade III and grade IV brainstem astrocytomas. Median survival for patients who did not receive surgery was 6 months, while the median survival for patients who did undergo surgery was 9 months (*p* = 0.055).

In our patient, we considered a stereotactic biopsy but the distinct delineation and ventrolateral location of the mass guided our decision to proceed with open biopsy, anticipating the potential for decompression and thus postoperative functional improvement, which would not be possible with a stereotactic biopsy. During the case, we decided that aggressive resection was not in the patient's best interest for multiple reasons. The firm rubbery texture of the lesion contributed to a temporary loss of MEPs intraoperatively during the debulking of the lesion. Additionally, the frozen section diagnosis of high-grade glioma reduced the enthusiasm for a potentially harmful large resection. Given the infiltrative nature typical of such gliomas, aggressive resection would not be curative and carried a high risk of causing additional morbidity.

### 3.4. Multimodality Therapy and Prognosis

As a group, adult malignant brainstem gliomas have an overall poor prognosis with a mean overall survival ranging from 11 to 17 months [3, 13], not very different from that of cerebral examples. In general, the administration of radiotherapy and concurrent temozolomide therapy has been shown to improve survival in patients with glioblastoma and is the current standard of care [45], but this has not been rigorously examined in the small population of adults with medullary glioblastomas.

Radiochemotherapy recommendations for patients with high-grade brainstem gliomas are less clear and study cohorts are typically small. Hundsberger et al. [2] described 13 patients with high-grade brainstem gliomas (grades III and IV). Seven patients were treated with radiation and concomitant temozolomide, three patients with radiation only, and 2 patients with chemotherapy only. The median radiation dose was 5760 cGy, with doses varying widely between 4500 and 6000 cGy. Median overall survival was 11.5 months.

Primary malignant brain tumors in the elderly (age > 65 years) carry a dismal prognosis compared to younger patients [46, 47]. The optimal strategy for adjuvant therapy in the elderly population is complicated by an increase in comorbidities. Babu et al. [4] reported on seven elderly patients (age > 60 years) with brainstem gliomas (grades III and IV, 2 had pontine lesions with medullary extention). Six patients survived to receive radiation therapy (dose range 5580–6120 cGy) with concurrent temozolomide (150–400 mg/m^2^ administered on days 1–5); all eventually recurred, received a variety of salvage therapies, and died. Median overall survival was 13.5 months (range 1.9–45.7 months).

Some patients may obtain clinical improvement after adjuvant therapy. In a study of 38 patients with brainstem glioma who underwent radiation treatment (mean total dose 5400 cGy), 50% experienced clinical improvement at 6 weeks [48]. Guillamo et al. [13] described only a 13% rate of clinical improvement after radiotherapy in 15 patients with grades III and IV brainstem lesions. A conservative radiotherapy dose of 5600 cGy was chosen for our patient. Approximately one month after radiation she experienced some improvement in RLE strength.

Modern evaluation and classification of gliomas are increasingly driven by molecular genetic analyses. Patients whose gliomas, even high-grade gliomas, have methylation of the promoter region of the gene for MGMT (which thereby suppresses expression of this DNA repair enzyme) have better response to radiation and alkylator chemotherapy and have longer survival. The presence in the tumor of a mutation of the IDH1 or, less commonly, IDH2 genes similarly is associated with better response to therapy and longer survival. On the other hand brainstem gliomas and other midline gliomas, mostly found in children, frequently are found to harbor mutations in the H3F3A gene (almost all the K27M mutation), and this is associated with a particularly poor prognosis and short survival. In this case the small amount of viable tumor tissue obtained for pathological analysis limited the testing done [49].

## 4. Conclusion

Glioblastoma of the medulla is rare. Biopsy can be performed relatively safely to provide definitive diagnosis to guide treatment, but long-term prognosis is poor.

## Figures and Tables

**Table 1:** Differential diagnosis of brainstem lesions.

Category	Diseases
Inflammatory	Autoimmune encephalitis
	Bickerstaff brainstem encephalitis
	CNS vasculitis
	Demyelination (multiple sclerosis)
	Neuromyelitis optica
	Neuro-Behçet
	Neurosarcoidosis
	Sjögren's syndrome with CNS involvement
	CLIPPERS (chronic lymphocytic inflammation with pontine perivascular enhancement responsive to steroids)

Neoplastic	Glioma
	Metastatic cancer
	CNS lymphoma
	Primitive neuroectodermal tumor
	Ependymoma
	Malignant histiocytosis

Infectious	Tuberculoma
	Pyogenic abscess

Paraneoplastic	Paraneoplastic brainstem encephalitis/rhombencephalitis

Vascular	Cavernous malformation
	Hematoma
	Arteriovenous malformation
	Cavernous angioma
	Ischemic infarct

**Table 2:** Summary of adult high-grade glioma case reports in the literature.

Reference	Year	Age	Lesion characteristics	Surgical treatment	Pathology	Multimodality therapy	Outcome
Our case	2014	69	Ventrolateral medulla	Biopsy via far lateral approach	GBM	Temozolomide and radiation	Died 11 months after diagnosis

Hundsberger et al. [2]	2014	48	Pons/medulla	Biopsy	US	US	US
		55	Medulla	Biopsy	US	US	US

Babu et al. [4]	2013	>60	Pons, MCP, medulla	US	US	US	US

Yoshikawa et al. [5]	2013	63	Ventral, diffuse medulla	None (diagnosis made at autopsy)	GBM	Temozolomide and radiation (tolerated for only 4 days)	Died 18 days after treatment

Chotai et al. [6]	2012	51	Dorsal, exophytic medulla	NTR via suboccipital approach	GBM	Temozolomide and radiation	19 months postsurgical survival

Lakhan and Harle [7]	2009	48	Diffuse, pons, medulla, cervical spine	None (diagnosis made at autopsy)	GBM	None	Died 4 weeks after presentation

Luetjens et al. [8]	2009	40	Dorsal, exophytic medulla	NTR via suboccipital approach	GBM	Temozolomide and radiation	Two years postsurgical survival

Shad et al. [9]	2005	28	Pons, medulla	Sx biopsy	AA	US	US

Kyoshima et al. [10]	2004	55	Dorsal, exophytic, medulla	GTR via suboccipital approach	GBM	Radiation for recurrence 1 year and 8 months postoperatively	Died 2 years and 3 months after surgery

Massager et al. [11]	2000	34	Medulla	US	AA	US	US
		37	Medulla	US	AA	US	US

Sahni et al. [12]	1987	24	Medulla	Sx biopsy	AA	Radiation	US
		27	Cervicomedullary junction	Sx biopsy	AA	Radiation	Died 1.5 years after surgery
		30	Medulla, 4th ventricle	Sx biopsy	AA	Radiation	US

US: unspecified.

AA: anaplastic astrocytoma.

Sx: Stereotactic.

GTR: gross total resection.

NTR: Near total resection.

MCP: middle cerebellar peduncle.

## Abbreviations

BAEPs: Brainstem auditory evoked potentials
EMG: Electromyography
CNS: Central nervous system
CSF: Cerebrospinal fluid
MRI: Magnetic resonance imaging
MLF: Medial longitudinal fasciculus
MEPs: Motor evoked potentials
SEER database: Surveillance, Epidemiology and End Results database
SSEPs: Somatosensory evoked potentials
WHO: World Health Organization.

## Additional Points

(i) The differential diagnosis for lesions of the ventrolateral medulla is extensive. (ii) Biopsy can be performed safely to provide a diagnosis and guide treatment. (iii) Despite adjuvant therapy, long-term prognosis remains poor.
